# Detraining and Anabolic-Androgenic Steroid Discontinuation Change Calcaneal Tendon Morphology

**DOI:** 10.3390/jfmk4010001

**Published:** 2018-12-21

**Authors:** Anderson José Santana Oliveira, Lívia Larissa Batista e Silva, Fabrício Reichert Barin, Elaine Cristina Leite Pereira, Heloisa Sobreiro Selistre-de-Araujo, Rita de Cássia Marqueti

**Affiliations:** 1 Faculty of Ceilândia, University of Brasília, Brasília, DF 72220-275, Brazil; 2 Departament of Physiological Sciences, Federal University of São Carlos, São Carlos, SP 13565-905, Brazil

**Keywords:** anabolic androgenic steroids (AAS), water jumping exercise, resistance training, detraining, extracellular matrix, tendon

## Abstract

Several side effects of anabolic-androgenic steroid (AAS) administration associated with training are reported in the biomechanical properties of the calcaneal tendon (CT) of rats. Thus, the aim of the present study is to evaluate the effects of the detraining and discontinuation of AAS administration on the CT morphology of rats submitted to exercise in water. Animals were divided into two groups (20/group): (1) Immediately after training (IA), and (2) Six weeks of detraining and AAS discontinuation (6W). The IA group included four subgroups: Sedentary (S), Trained (T), Sedentary with AAS administration (SAAS), and trained with AAS administration (TAAS). The 6W group included four subgroups: Sedentary (6W-S), six weeks of detrained (6W-T), six weeks of sedentary with AAS discontinuation (6W-SAAS), and six weeks of detrained with AAS discontinuation (6W-TAAS). Data show significant reduction in adipose cells volume density (Vv%) in the distal CT in 6W-TAAS group, indicating that training can exert a positive effect on the tendon. The 6W-SAAS group exhibited increased adipose cells Vv% in the distal region, compared with the W6-S and W6-T groups. A decrease in tendon proper cells Vv% and in peritendinous sheath cells Vv% of proximal and distal regions was also observed. In 6W-TAAS group showed increase in adipose cells, blood vessels, peritendinous sheath cells, and tendon proper cells Vv% in the distal region of the CT. The vertical jumps in water were not able to protect CT regions from the negative effects of AAS discontinuation for six weeks. However, after detraining and AAS discontinuation, many protective factors of the mechanical load in the long-term could be observed.

## 1. Introduction

The calcaneal tendon (CT) is the thickest and strongest tendon in the human body. The CT consists of fibers originating from two muscles: The soleus muscle (SOL), which lays deep, and the gastrocnemius muscle, which lays superficially. The latter is composed of two heads—the lateral head of the gastrocnemius muscle (GL) and the medial head of the gastrocnemius muscle (GM), from each of which originates an independent subtendon [1]. Tendons consist of dense fibrous connective tissue that attaches muscles to bones. They are composed of a large density of collagen fibers and fibroblasts (tenocytes) embedded in a unique extracellular matrix (ECM) [2]. The main function of tendons is to transfer the contractile forces generated by the muscles to the bones, generating movement [3].

It is well-documented that mechanical loading (e.g., exercise) increases the expression and secretion of several regulatory factors of tenocyte proliferation, ECM remodeling, and collagen synthesis in tendons [4,5,6,7]. It is generally presumed that the increased secretion of these growth factors and enzymes is responsible for the development of exercise-induced adaptations, which include an increased tendon cross-sectional area, tendon stiffness, and collagen crosslinking [2,8,9]. Thus, it is clear that physiological loads influence tendon cells, producing cellular signals that lead to positive adaptations to the tissue.

On the other hand, a systematic review investigated the effects of training interruption on tendon mechanobiology, indicating that detraining (four weeks of no exercise in animals) causes rearrangement in the collagen fiber, increasing collagen type III and reducing collagen type I, causing a loss of resistance to tension, increasing rigidity and the risk of rupture in the entheses [10]. In addition, detraining leads to a reduction in collagen type I and III synthesis and tenocyte activity, despite the matrix metalloproteinases (MMPs) synthesized during the training phase remaining active, assisting tissue remodeling [10,11].

Moreover, in sports, particularly in competitive athletes, the use of anabolic-androgenic steroids (AAS) has already become a chronic practice due to the continuous search for better performance [12]. AAS can cause various adverse effects, such as an elevated risk of tendon rupture [13,14], likely due to degenerative changes in the tendon, and disorganized collagen fibers, including dysplastic fibrils with a clear disruption of the fibril interface [15].

A number of harmful side effects of AAS administration associated with training in rats are reported in the biomechanical properties of the CT, superficial flexor tendon (SFT), and deep flexor tendon (DFT), characterized by a less flexible and weaker tendon [16]. Histological alterations in these three tendons have also been observed and are associated with a reduction in blood vessel volume density (Vv%), increased adipose cell volume density, the presence of synovial-like cells, and a reduction in the hydroxyproline content in some tendon regions [17]. Taken together, these findings suggest significant concern, as AAS seems to reverse the beneficial effects of exercise. However, it is not known whether discontinuation of AAS reverses the negative effects of these drugs on tendons.

Following this information, our hypothesis is that AAS administration associated with detraining would induce adverse effects on the CT, which would not be reversed six weeks after stopping AAS administration. Outstandingly, the negative effects caused by AAS will be considered in this study, such as cellular and structural alterations. On the other hand, we hypothesize that the mechanical demand inherent to exercise will lead to cellular and structural benefits, which may not remain after six weeks of detraining. Thus, the aim of the present study was to analyze the effects of detraining and the discontinuation of AAS administration on adipose cells, blood vessels, peritendinous sheath cells, and tendon proper cells in the calcaneal tendon of rats submitted to vertical jump exercise in water.

## 2. Materials and Methods

### 2.1. Animals

Forty male Wistar rats (Ratus novergicus albinus) weighing 200 ± 17 g were used (eight weeks old). The research protocol received approval from the Ethics Committee on Animal Experimentation from the Federal University of Sao Carlos, SP, Brazil (4 April 2006 (UFSCar/PPG-CFS), CEEA 004/2006), and all procedures were conducted in accordance with the guidelines of the Brazilian College for Animal Experimentation (COBEA). The animals were housed under a constant temperature (22 ± 2 °C) and light cycle (12:12 h light-dark cycle) with free access to standard rat food and tap water.

### 2.2. Experimental Groups

**Figure 1:**
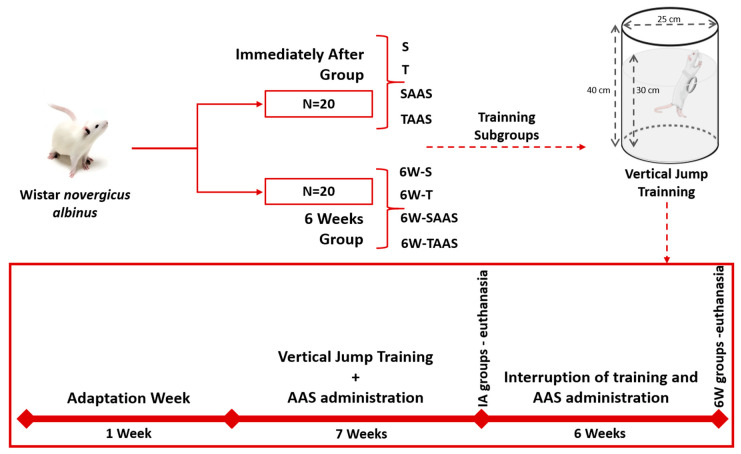
Experimental design. Schematic illustration of the methodological sequence followed in the study. Immediately after (IA) groups: Sedentary (**S**), trained (**T**), sedentary with anabolic-androgenic steroid (AAS) administration (**SAAS**), and trained AAS administration (**TAAS**). The six weeks—6W groups: Sedentary (**6W-S**), 6-weeks of detrained (**6W-T**), 6-weeks of sedentary with AAS discontinuation (**6W-SAAS**), and 6-weeks of detrained with AAS discontinuation (**6W-TAAS**).

The animals were randomly divided into two main groups: Immediately after—IA and six weeks—6W, with 20 animals per group (Figure 1). The IA-group was composed of the following subgroups (5 animals/subgroup): Sedentary (S), trained (T), sedentary with anabolic androgenic steroid administration (SAAS), and trained with anabolic androgenic steroid administration (TAAS).

The 6W-group was composed of the following subgroups (five animals/subgroup): Plus six weeks of sedentary (6W-S), plus six weeks of detrained (6W-T), plus six weeks of sedentary with AAS discontinuation (6W-SAAS), and plus six weeks of detrained with AAS discontinuation (6W-TAAS). The 6W-groups followed the exact same conditions as the IA groups until euthanasia of the IA animals (Figure 1). 

### 2.3. Anabolic Androgenic Steroid Administration

The rats (SAAS and TAAS groups) were submitted to an AAS administration protocol, in which they received 5 mg/kg of body mass (supraphysiological dose) of nandrolone decanoate (Deca-Durabolin^®^, Organon do Brasil, São Paulo, Brazil) administered subcutaneously in their backs twice a week. This is considered a supraphysiological dose, like the doses used by athletes [18]. The experimental groups without AAS treatment (S and T) received the vehicle only (peanut oil with benzyl alcohol). The treatment started in the first training week and continued for seven weeks.

### 2.4. Training Protocol

Adaptation week: To reduce stress, the animals were adapted to water in the pretraining week. This adaptation consisted of weight lifting sessions (50% body weight load), once a day, five days per week, in water at 30 ± 2 °C. The training was induced by the instinctive reactions of rats submitted to a jump exercise protocol in a plastic tube (25 cm diameter and 40 cm length) with water at 30 ± 2 °C. The overload was attached to the animal’s chest by means of a vest fitted to its body. The numbers of sets [2,3,4] and repetitions [5,6,7,8,9,10] were adjusted daily and increased gradually. All sessions were performed in the afternoon after 4 p.m.

Vertical jump protocol: After the adaptation week, the animals were submitted to the experimental jump protocol, which consisted in the first training week of: 4 sets of 10 jumps with a 30 s rest period between sets and overload of 50% of body weight. In the next six weeks, the training protocol continued with the same number of sets (4 sets), jumps (10), and rest intervals (30 s between sets), but with increased overload (5% increase per week), reaching 80% of body weight in the final week.

### 2.5. Histological Preparation

In week seven, the training and steroid administration were interrupted and the IA group was euthanized. After six weeks of detraining and interruption of steroid administration, the 6Ws group was euthanized. This procedure was performed through an overdose of xylazine and ketamine in the intraperitoneal region (12 mg/kg of body weight and 95 mg/kg of body weight, respectively).

After extracting the calcaneus tendons from the animals, they were fixed by immersion in 4% formaldehyde in phosphate buffered saline for 24 h, washed with distilled water, dehydrated in 70% ethanol, and embedded in glycol methacrylate resin (Leica Microsystems, Heidelberg, Germany). Two-micrometer sections were obtained from the tendons using glass knives and were stained with hematoxylin-eosin (HE) (Nuclear, São Paulo, Brazil).

### 2.6. Histomorphometric Analysis 

Histomorphometric analyses were performed using a microscope (Olympus BV51) linked to a digital camera, SV Micro Sound Vision (Preston South, Australia), to capture images at 20× magnification; 10 nonconsecutive digital images per area were obtained (peritendinous and tendon proper). The images were analyzed using Photoshop software (Adobe Systems Inc., San Jose, California, USA). A planimetry system with a translucent Weibel grid [16,19] superimposed over each image was used to determine the volume density (Vv%) of the adipose cells, blood vessels (blood vessel lumen, endothelial cells, and perivascular sheath), peritendinous sheath cells (other cells), and tendon proper cells (fibroblasts and fibrochondrocyte-like cells). The stereology was performed by counting the structures when they coincided with the planimetry system points. After counting, the percentages of the structures of each region and sheath were calculated. The calculus of the peritendinous sheath was different, as it depended on how many points coincided with the tendon proper sheath. To calculate Vv%, the number of points coincident with the structures was multiplied by 100, then divided by the total number of grid points.

### 2.7. Statistical Analysis

Statistical evaluation was performed initially using the Kolmogorov-Smirnov test to assess data normality, then the ANOVA two-way test, followed by Turkey analysis to compare the effects between interventions. A nonpaired Student’s t-test was used to determine the significance of the differences between the IA and 6W groups. The data are presented as mean ± standard error of the mean. A significance level of 5% (*p* < 0.05) was adopted and the Statistica 7.0 software package (Stat. Soft. Inc., Tusa, OK, USA) was used for all analyses.

## 3. Results

### 3.1. Histomorphometry Immediately After Seven Weeks of Training (IA)

**Figure 2:**
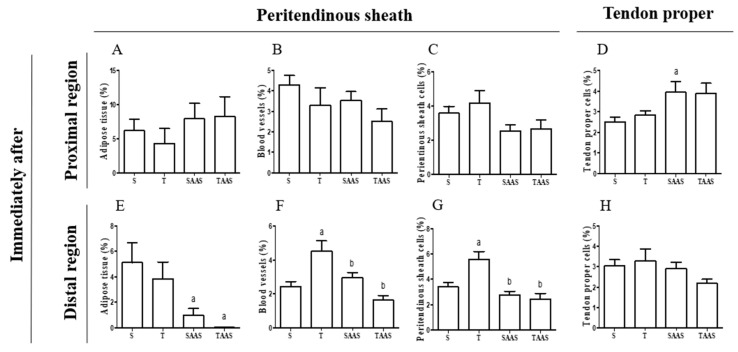
Volume density variation (Vv%) in structural elements found in the proximal and distal regions of the calcaneal tendon (CT) in the IA group and subgroups. Adipose tissue (**A**,**E**), Blood vessels (**B**,**F**), Peritendinous sheath cells (**C**,**G**), Tendon proper cells (**D**,**H**). Values are expressed as means ± standard error of the mean (*p* ≤ 0.05). (**a**) Significant difference vs. S subgroup, (**b**) significant difference vs. T subgroup.

**Figure 3:**
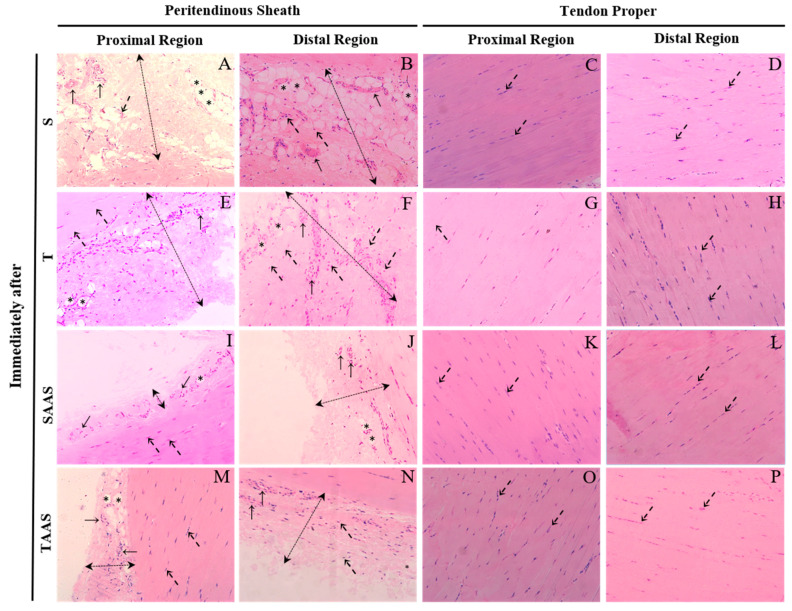
Longitudinal sections of the proximal and distal regions of the CT in the IA group stained with hematoxylin-eosin. Sedentary subgroup (**S**): In the peritendinous sheath (dashed double-head arrow), the proximal (**A**) and distal (**B**) regions show blood vessels (arrows), cells (dashed arrows), and adipose cells (asterisks). In the tendon proper, the proximal (**C**) and distal (**D**) regions show cells (dashed arrows). Trained subgroup (**T)**: In the peritendinous sheath (dashed double-head arrow), the proximal (**E**) and distal (**F**) regions show blood vessels (arrows), cells (dashed arrows), and adipose cells (asterisks). In the tendon proper, the proximal (**G**) and distal (**H**) regions show cells (dashed arrows). Sedentary with AAS administration (**SAAS**): In the peritendinous sheath (dashed double-head arrow), the proximal (**I**) and distal (**J**) regions show blood vessels (arrows), cells (dashed arrows), and adipose cells (asterisks). In the tendon proper, the proximal (**K**) and distal (**L**) regions show cells (dashed arrows). Trained with AAS administration (**TAAS**): In the peritendinous sheath (dashed double-head arrow), the proximal (**M**) and distal (**N**) regions show blood vessels (arrows), cells (dashed arrows), and adipose cells (asterisks). In the tendon proper, the proximal (**O**) and distal (**P**) regions show cells (dashed arrows). Original magnification, 400×. Scale bars 20 µm.

Seven weeks of training increased blood vessels Vv% and peritendinous sheath cells in the distal region of the CT when compared with the control group (S) (Figure 2F,G and Figure 3F). Additionally, in the T group, tenocytes showed an oval shape, and were aligned to the collagen bundles in the tendon proper of the proximal region. AAS treatment increased cells Vv% in the proximal region of the tendon proper (Figure 2D) and promoted a reduction in the adipose cells Vv% in the distal region compared to the S group (Figure 1E). There was also a reduction in blood vessels Vv% and peritendinous sheath cells in the distal region (Figure 2F,G) in comparison with the T group.

The administration of AAS associated with the training promoted a reduction in adipose cells Vv% in the distal region of the CT in comparison with the S group (Figure 2E and Figure 3N). There was also a decrease in peritendinous sheath cells Vv% and blood vessels Vv% in the distal region in comparison with the T group (Figure 2F,G and Figure 3N). No significant differences were observed between experimental groups regarding tendon proper cells Vv% in the distal region of the CT (Figure 2H and Figure 3O,P).

### 3.2. Histomorphometry after 6 Weeks of Detraining (6W)

**Figure 4:**
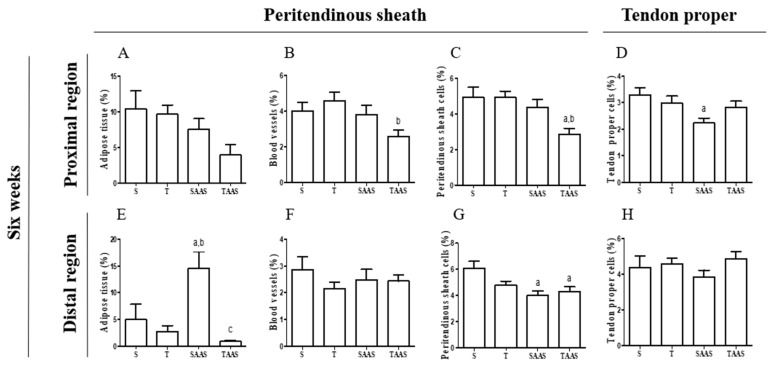
Volume density variation (Vv%) in structural elements found in the proximal and distal regions of the CT in the 6W group and subgroups. Adipose tissue (**A**,**E**), Blood vessels (**B**,**F**), Peritendinous sheath cells (**C**,**G**), Tendon proper cells (**D**,**H**). Values are expressed as means ± standard error of the mean (*p* ≤ 0.05). (**a**) Significant difference vs. 6W-S subgroup, (**b**) significant difference vs. 6W-T subgroup, (**c**) significant difference vs. 6W-SAAS subgroup.

**Figure 5:**
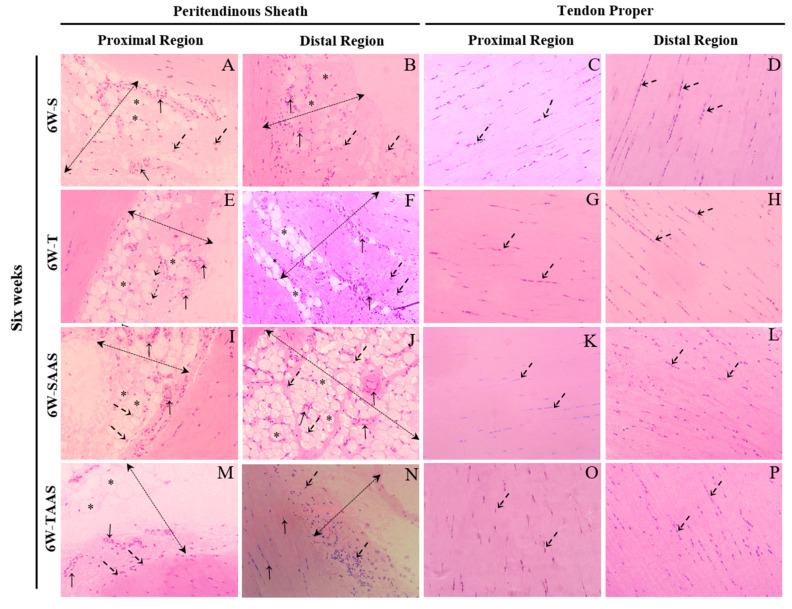
Longitudinal sections of the proximal and distal regions of the CT in the 6W group stained with hematoxylin-eosin. 6W-S: In the peritendinous sheath (dashed double-head arrow), the proximal (**A**) and distal (**B**) regions show blood vessels (arrows), cells (dashed arrows), and adipose cells (asterisks. In the tendon proper, the proximal (**C**) and distal (**D**) regions show cells (dashed arrows). 6W-T: In the peritendinous sheath (dashed double-head arrow), the proximal (**E**) and distal (**F**) regions show blood vessels (arrows), cells (dashed arrows), and adipose cells (asterisks). In the tendon proper, the proximal (**G**) and distal (**H**) regions show cells (dashed arrows). 6W-SAAS: In the peritendinous sheath (dashed double-head arrow), the proximal (**I**) and distal (**J**) regions show blood vessels (arrows), cells (dashed arrows), and adipose cells (asterisks). In the tendon proper, the proximal (**K**) and distal (**L**) regions show cells (dashed arrows). 6W-TAAS: In the peritendinous sheath (dashed double-head arrow), the proximal (**M**) and distal (**N**) regions show blood vessels (arrows), cells (dashed arrows), and adipose cells (asterisks). In the tendon proper, the proximal (**O**) and distal (**P**) regions show cells (dashed arrows). Original magnification, 400×. Scale bars 20 µm.

The T6 group did not demonstrate differences in adipose cells, blood vessels, peritendinous sheath cells, or tendon proper cells Vv% in their distal and proximal regions, compared to the S6 group (Figure 4A–H and Figure 5E–H).

The 6W-SAAS group exhibited significantly greater adipose cells Vv% in the distal region, in comparison with the 6W-S and 6W-T groups (Figure 4E). A reduction was also observed in tendon proper cells Vv% and peritendinous sheath cells Vv% of proximal and distal regions, respectively, in comparison to the 6W-S group (Figure 4D,G, respectively). In addition, the tenocytes of tendon proper cells in the distal region of the CT seemed to be scattered and less aligned than other groups (Figure 5L).

Additionally, the data show a significant reduction in adipose cells Vv% in the CT distal region of the 6W-TAA group when compared to the 6W-SAAS group. Blood vessels Vv% decreased in the 6W-TAA group only in the proximal region of the CT when compared with the 6W-T group (Figure 4B and Figure 5M). A reduction was also found in peritendinous sheath cells Vv% of the CT proximal region when compared with the 6W-Sand 6W-T groups (Figure 4C and Figure 5M). Peritendinous sheath cells Vv% also decreased in the CT distal region in comparison with the 6W-S group (Figure 4G and Figure 5N).

### 3.3. Temporal Comparison between IA and 6W Groups

In order to understand the consequences of six weeks without training and/or AAS administration, comparisons between each similar experimental group were performed and are shown in Table 1.

None of the evaluated variables demonstrated significant differences between the S and 6W-S groups, except for peritendinous sheath cells Vv% in the distal regions of the CT, which increased in the 6W-S group compared to the S group. Regarding vertical jumping, after six weeks of detraining, a significant increase in adipose cells Vv% was observed in the proximal region, followed by a reduction in blood vessels Vv% and an increase in tendon proper cells Vv% in the distal region.

AAS discontinuation facilitated a significant increase in adipose cells Vv% in the distal region, as well as increased peritendinous sheath cells Vv% in both proximal and distal regions of the CT and promoted a reduction in tendon proper cells Vv% in the proximal region. Finally, detraining associated with androgenic anabolic steroid discontinuation in the 6W-TAAS group promoted an increase in adipose cells, blood vessels, peritendinous sheath cells, and tendon proper cells Vv% in the distal region of the CT. On the other hand, there was a reduction in tendon proper cells Vv% in the proximal region of the CT.

## 4. Discussion

This study evaluated the effects of detraining and AAS discontinuation on histomorphological aspects of the peritendinous sheath and tendon proper in both proximal and distal regions of the CT. The main findings suggest that vertical jumping in water was not able to protect the CT from the negative effects of nandrolone decanoate discontinuation for six weeks. 

### 4.1. Training, AAS, and AAS Associated with Training Effects

Training promoted increased peritendinous sheath cells Vv% in response to vertical jumping only in the distal region of the CT. This response can be explained by increased tissue demand, enhanced connective tissue turnover, and ECM proteins, due to the increase in muscle contraction force, which promotes mechanical stimulation for collagen synthesis [20], an upregulation of stress-responsive cytokines and growth factors (e.g., insulin- like growth factor, transforming growth factor-β-1, and interleukin-6) [4,21], and increased mechanotransduction stimuli in the tendon cells to increase collagen synthesis, independent of short- or long-term exposure, which modifies tendon extracellular matrix and morphology [22].

Additionally, in the current study, the training was effective for increasing vascularization in the distal region of the CT. Tendons have poor vascularization. However, the vessels are important for ensuring normal cell functions and tissue repair [23]. Other studies have also observed a similar response regarding increased tendon vascularization, during or after exercise periods [17,24].

On the other hand, we found decreased cellularity and vascularization in the peritendinous sheath in the distal region when comparing both AAS groups to the trained group. Using the same experimental model and training time as the current study, another research demonstrated an increase in peritendinous cellularity [17], unlike our data. Despite this, using AAS for short or long periods can induce abnormalities in collagen distribution [25] and remodeling [5], which may interfere in fibroblast proliferation and induce cell death [26]. Thus, it is possible that the treatment with AAS in our study induced similar effects. This hypothesis remains to be further investigated.

Tendon morphological properties were negatively affected when training was associated with AAS administration, mainly in relation to the decreased vascularization and cellularity of the peritendinous sheath in the distal region, when compared to the T group. In general, many studies have shown that this combination (AAS plus training) can modify ECM properties in tendons, skeletal muscle, and cardiac muscle [5,6,16,17], and alter the key gene expressions responsible for tendon integrity [27], adapting tendon biomechanics, and making it more inclined to failure [15,16]. In the present study, an unexpected effect caused by significant adipose cell reduction in the distal region of SAAS and TAAS groups was observed. The probable explanation for this is the enhanced lipolytic activity and inhibited adipocyte differentiation caused by AAS [28]. 

### 4.2. AAS Discontinuation Effects

AAS discontinuation prompted increased adipose cells at high levels in the distal region of the 6W-SAAS, and peritendinous sheath and tendon proper cell reduction. AAS binds to the androgen receptor (AR) in target tissues to exert its androgenic and anabolic effects. The AR regulates the transcription of target genes that control the accumulation of DNA required for muscle growth [29,30]. Physiological influences of AAS induce increases in protein synthesis in skeletal muscle, promoting an increase in muscle mass [30]. However, our study shows that AAS discontinuation in the sedentary subgroup exerted an exacerbated negative effect (rebound) on tendons, increasing adipose cells and reducing cellularity. 

Several bioactive peptides (chemerin, leptin, adiponectin, and others) are released by adipocytes, and influence tendon structure by means of negative activities on mesenchymal cells [31]. Tendon-derived stem cells (TDSCs) are similar to other multipotent stem cells. They are able to self-renew and present adipogenic, chondrogenic, and osteogenic differentiation potentials [32]. 

Increased levels of prostaglandin E_2_ (PGE_2_), a major inflammatory mediator in tendons, decreased proliferation of TDSCs in tendons, inducing differentiation of TDSCs into adipocytes and osteocytes in vitro [33]. Fine control of tenocyte differentiation is necessary to maintain tendon homeostasis [34,35], and it is possible that discontinuation of AAS treatment promoted an imbalance in tendon homeostasis, which was prevented by exercise in the present study. 

Furthermore, Scott et al. [31] showed that high levels of adipocytes are an important factor in the development of tendinopathies, as they contribute to structural damage in the tissue, leading to a pattern of function loss (degeneration) [36] characterized by disorganized collagen deposition and extravagant scar formation during healing, which may result from excessive myofibroblast differentiation [37]. 

This culminates in poor tissue capacity to respond to large demands imposed [38]. Clusters of adipocytes were found in ruptured tendons of humans within spaces between the collagen fibers. The adipocytes appear to disrupt the continuity of the collagen fibers and bundles, diminishing the tensile strength of the tendon [39].

As previously mentioned, a decrease in tendon proper cells was observed in the 6W-SAAS subgroup. Studies have shown that high levels of apoptosis in tendons may be one of the explanations for structural alterations, leading to tendinopathies [40,41,42]. Apoptosis, when natural, is a physiological process where programmed cell death occurs and can be mediated by several factors, which are essential for the preservation of tissue homeostasis and elimination of damaged cells [41]. However, the abrupt reduction in cells through apoptotic processes, which have repercussions on hypocellular areas, evidenced in some histological studies, may influence the collagen synthesis rate and organization of tendon ECM, impairing the repair of injured tendons [42]. It seems clear that our findings indicate a nonfunctional tendon response after AAS discontinuation, possibly due to alteration in the tendon remodeling process. 

### 4.3. Effects of AAS Discontinuation and Detraining

The combination of AAS discontinuation and detraining showed a reduction in blood vessels and peritendinous sheath cells in proximal and distal regions. These results indicate that the negative effects prompted by ASS remain, since the same side effects were observed immediately after the period. For example, a combination of training and AAS administration inhibited the messenger RNA (mRNA) of vascular endothelial growth factor (VEGF), whereas jump training alone increased the mRNA of VEGF [43]. VEGF induces invasion of endothelial cells and vessels in hypovascularized tissue [44]. Thus, it is clear that anabolic steroids, even after six weeks of discontinuation, block some of the beneficial effects of exercise. However, contrary to the large increase in adipose tissue previously discussed and shown in the SAAS group, the addition of training inhibited the adipose increase, demonstrating a probable protective factor of training mechanics in the long-term.

### 4.4. Temporal Comparison between IA and 6W Groups

As mentioned above, this study was also designed to analyze comparisons between IA and 6W groups, observing the temporal alterations that occurred. In the sedentary control group (S), the content of peritendinous sheath cells increased after six weeks in sedentary animals. It is possible that over the time of experimentation (six weeks), the animals increased their muscle mass, which could generate mechanical stimulus to tendon tissue, culminating in positive cellular alterations.

Regarding detraining effects (trained and six weeks of detraining group), the current results show that a six week untrained period was able to revert the resistance training benefits, restoring peritendinous sheath cells and blood vessels to basal levels. This may have occurred in response to the lack of tissue demand occasioned by training discontinuation, which is a physiologically expected response [45]. The time required for the maintenance of benefits obtained from exercise on tendons after training discontinuation remains controversial. Decreases or interruptions in habitual activity level (training interruption) result in partial or complete loss of anatomical, physiological, and performance-induced adaptations, which vary quantitatively and qualitatively depending on the gap time [46,47]. Several studies in vitro and in vivo have shown decreases in tenocyte, synthetic, and metabolic activity, in tendon morphology, and in its enthesis, due to treadmill training discontinuation [11,48,49]. These alterations involved decreases in proteoglycan content and loss of collagen fiber organization, with an increase in collagen III and a decrease in collagen I, resulting in less resistance to stress and a related increased risk of tendon rupture [10]. However, increases were observed in the tendon proper cells after six weeks of detraining in comparison to the IA group. This suggests migration of peritendinous sheath cells to the inner tendon as a benefit of training.

In the sedentary and treated group (SAAS), AAS discontinuation resulted in an exacerbated increase in adipocytes, followed by an increase in peritendinous sheath cells in the distal and proximal regions and tendon proper cell reduction. The AAS discontinuation stimulus promotes effects contrary to those observed during the time of use. As mentioned above, an increase in adipocytes harms the capabilities of functional tendons and evidences the negative effects of AAS, even after six weeks of interruption. However, the increase in cells in the peritendinous sheath (proximal and distal), may be explained as a possible attempt to restore homeostasis after AAS deprivation.

**Figure 6:**
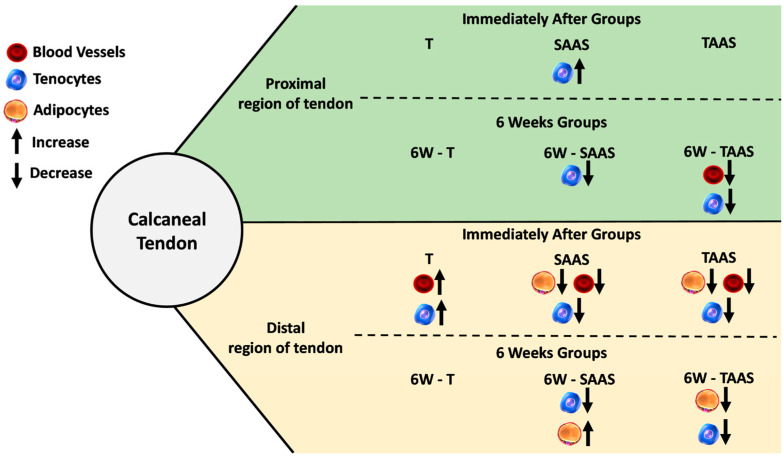
Overview of main results acquired during this study, showing that AAS promoted harmful effects on tendon tissue morphology mainly in distal region.

Despite reasonable support by reports indicating that AAS may counteract the irreparable structural alterations in the CT after interruption, no studies including molecular and functional analyses have been performed. In order to provide potential evidence, these analyses are required in additional studies. The results suggest that during AAS use, cellular receptors could be saturated and hamper cellular differentiation. However, following the drug deprivation, the peritendinous sheath cells exacerbate the proliferation comparison between the 6W-TAAS and TAAS groups (IA) showing that, six weeks after AAS deprivation, the tendon attempts to reestablish tissue homeostasis. Training exerts a delayed effect on the tendon, similar to “memorizing an event,” interpreted by cell signaling even after detraining and AAS discontinuation. Thus, both vascularization and cell content (tissue-dependent elements) respond to loading mechanisms. Finally, the Figure 6 bellow shows the main results achieved in this study.

## 5. Conclusions

In conclusion, our findings support that after detraining and AAS discontinuation, the beneficial effects of training are no longer evident. However, the vascularization and cellularity were restored, while the adipose cells were retained in a low amount, indicating that training was able to protect the CT from the detrimental effects of AAS. Together, these results sustain that there is a protective factor related to long-term mechanical load in CT even after six weeks of detraining.

## Figures and Tables

**Table 1:** Temporal comparison of volume density (Vv%) between IA and 6W groups.

		S		T		SAAS		TAAS	
		IA (Vv%) Mean (SEM)	6W (Vv%) Mean (SEM)	IA (Vv%) Mean (SEM)	6W (Vv%) Mean (SEM)	IA (Vv%) Mean (SEM)	6W (Vv%) Mean (SEM)	IA (Vv%) Mean (SEM)	6W (Vv%) Mean (SEM)
**Proximal Region**	Adipocytes	6.19 (11.13)	10.42 (14.05)	4.32 (10.11)	9.64 (9.33) *	7.92 (13.49)	7.55 (11.18)	8.24 (13.36)	3.93 (7.97)
	Blood Vessels	4.29 (3.05)	4.00 (2.68)	3.29 (4.06)	4.59 (3.39)	3.53 (2.54)	3.80 (3.95)	2.51 (2.77)	2.59 (1.93)
	Peritendinous Sheath Cells	3.59 (2.63)	4.92 (2.68)	4.17 (3.46)	4.92 (2.45)	2.55 (2.08)	4.38 (3.23) **	2.68 (2.32)	2.86 (1.75)
	Tendon Proper Cells	2.31 (1.15)	2.79 (0.98)	2.84 (1.26)	2.98 (1.70)	3.95 (2.81)	2.24 (1.06) **	3.89 (1.99)	2.83 (1.20) *
**Distal Region**	Adipocytes	5.12 (10.88)	4.97 (14.01)	4.84 (9.50)	2.75 (7.35)	0.97 (3.76)	14.58 (21.35) ***	0.04 (0.19)	0.87 (1.92) *
	Blood Vessels	2.43 (2.01)	2.86 (2.38)	4.51 (3.59)	2.16 (1.62) ***	2.95 (2.11)	2.49 (2.68)	1.63 (1.40)	2.44 (1.82) *
	Peritendinous Sheath Cells	3.40 (2.38)	6.10 (2.56) ***	5.57 (3.68)	4.80 (2.02)	2.76 (1.98)	4.00 (2.47) **	2.45 (2.20)	4.31 (2.84) **
	Tendon Proper Cells	2.56 (1.09)	2.97 (0.81)	3.29 (2.63)	4.59 (2.40) *	2.92 (1.62)	3.84 (1.91)	2.19 (1.10)	4.87 (2.77) ***

Values are expressed as means ± standard error of the mean. Nonpaired Students’ *t*-test was used to determine the significance of the differences between IA and 6W subgroups. *, *p* ≤ 0.05; **, *p* ≤ 0.01; ***, *p* ≤ 0.05. Sedentary (S), trained (T), sedentary with anabolic-androgenic steroid (AAS) administration (SAAS), and trained AAS administration (TAAS).

## References

[1] Ballal M.S., Walker C.R., Molloy A.P. (2014). The anatomical footprint of the Achilles Tendon. Bone Jt. J..

[2] Subramanian A., Schilling T.F. (2015). Tendon development and musculoskeletal assembly: Emerging roles for the extracellular matrix. Development.

[3] Thomopoulos S., Genin G.M., Galatz L.M. (2010). The development and morphogenesis of the tendon-to-bone insertion—What development can teach us about healing. J. Musculoskelet. Neuronal Interact..

[4] Heinemeier K.M., Olesen J.L., Haddad F., Langberg H., Kjaer M., Baldwin K.M., Schjerling P. (2007). Expression of collagen and related growth factors in rat tendon and skeletal muscle in response to specific contraction types. J. Physiol..

[5] Marqueti R.C., Parizotto N.A., Chriguer R.S., Perez S.E.A., Selistre-de-Araujo H.S. (2006). Androgenic-anabolic steroids associated with mechanical loading inhibit matrix metallopeptidase activity and affect the remodeling of the achilles tendon in rats. Am. J. Sports Med..

[6] Marqueti R.C., Prestes J., Paschoal M., Ramos O.H.P., Perez S.E.A., Carvalho H.F., Selistre-de-Araujo H.S. (2008). Matrix metallopeptidase 2 activity in tendon regions: Effects of mechanical loading exercise associated to anabolic-androgenic steroids. Eur. J. Appl. Physiol..

[7] Maeda E., Hagiwara Y., Wang J.H.C., Ohashi T. (2013). A new experimental system for simultaneous application of cyclic tensile strain and fluid shear stress to tenocytes in vitro. Biomed. Microdevices.

[8] Kongsgaard M., Reitelseder S., Pedersen T.G., Holm L., Aagaard P., Kjaer M., Magnusson S.P. (2007). Region specific patellar tendon hypertrophy in humans following resistance training. Acta Physiol..

[9] Carroll C.C., Whitt J.A., Peterson A., Gump B.S., Tedeschi J., Broderick T.L. (2012). Influence of acetaminophen consumption and exercise on Achilles tendon structural properties in male Wistar rats. AJP Regul. Integr. Comp. Physiol..

[10] Frizziero A., Salamanna F., Della Bella E., Vittadini F., Gasparre G., Nicoli Aldini N., Masiero S., Fini M. (2016). The Role of Detraining in Tendon Mechanobiology. Front Aging Neurosci..

[11] Frizziero A., Fini M., Salamanna F., Veicsteinas A., Maffulli N., Marini M. (2011). Effect of training and sudden detraining on the patellar tendon and its enthesis in rats. BMC Musculoskelet. Disord..

[12] Pope H.G., Wood R.I., Rogol A., Nyberg F., Bowers L., Bhasin S. (2014). Adverse Health Consequences of Performance-Enhancing Drugs: An Endocrine Society Scientific Statement. Endocr. Rev..

[13] Kanayama G., Deluca J., Meehan W.P., Hudson J.I., Isaacs S., Baggish A., Weiner R., Micheli L., Pope H.G. (2015). Ruptured tendons in anabolic-androgenic steroid users. Am. J. Sports Med..

[14] Jones I.A., Togashi R., Hatch G.F.R., Weber A.E., Vangsness C.T. (2018). Anabolic steroids and tendons: A review of their mechanical, structural, and biologic effects. J. Orthop. Res..

[15] Tsitsilonis S., Panayiotis C.E., Athanasios M.S., Stavros K.K., Ioannis V.S., George A., Konstantinos F., Despina P.N., Aristides Z.B. (2014). Anabolic androgenic steroids reverse the beneficial effect of exercise on tendon biomechanics: An experimental study. Foot Ankle Surg..

[16] Marqueti R.C., Prestes J., Wang C.C., Ramos O.H.P., Perez S.E.A., Nakagaki W.R., Carvalho H.F., Selistre-de-Araujo H.S. (2011). Biomechanical responses of different rat tendons to nandrolone decanoate and load exercise. Scand. J. Med. Sci. Sports..

[17] Marqueti R.C., Paulino M.G., Fernandes M.N., de Oliveira E.M., Selistre-de-Araujo H.S. (2014). Tendon structural adaptations to load exercise are inhibited by anabolic androgenic steroids. Scand. J. Med. Sci. Sports.

[18] Pope H.G., Katz D.L. (1988). Affective and psychotic symptoms associated with anabolic steroid use. Am. J. Psychiatr..

[19] Weibel E.R. (1969). Stereological Principles for Morphometry in Electron Microscopic Cytology. Int. Rev. Cytol..

[20] Kjær M., Langberg H., Heinemeier K., Bayer M.L., Hansen M., Holm L., Doessing S., Kongsgaard M., Krogsgaard M.R., Magnusson S.P. (2009). From mechanical loading to collagen synthesis, structural changes and function in human tendon. Scand. J. Med. Sci. Sports.

[21] Olesen J.L., Heinemeier K.M., Gemmer C., Kjær M., Flyvbjerg A., Langberg H. (2007). Exercise-dependent IGF-I, IGFBPs, and type I collagen changes in human peritendinous connective tissue determined by microdialysis. J. Appl. Physiol..

[22] Heinemeier K.M., Kjaer M. (2011). In vivo investigation of tendon responses to mechanical loading. J. Musculoskelet. Neuronal Interact..

[23] Benjamin M., Kaiser E., Milz S. (2008). Structure-function relationships in tendons: A review. J. Anat..

[24] Malheiro O.C.D.M., Giacomini C.T., Justulin L.A., Delella F.K., Dal-Pai-Silva M., Felisbino S.L. (2009). Calcaneal tendon regions exhibit different MMP-2 activation after vertical jumping and treadmill running. Anat. Rec..

[25] Michna H. (1987). Tendon injuries induced by exercise and anabolic steroids in experimental mice. Int. Orthop..

[26] Taguchi T., Kubota M., Saito M., Hattori H., Kimura T., Marumo K. (2016). Quantitative and qualitative change of collagen of achilles tendons in rats with systemic administration of glucocorticoids. Foot Ankle Int..

[27] Marqueti R.d.C., Heinemeier K.M., Durigan J.L.Q., De Andrade Perez S.E., Schjerling P., Kjaer M., Carvalho H.F., Selistre-de-Araujo H.S. (2012). Gene expression in distinct regions of rat tendons in response to jump training combined with anabolic androgenic steroid administration. Eur. J. Appl. Physiol..

[28] Flück M., Ruoss S., Möhl C.B., Valdivieso P., Benn M.C., von Rechenberg B., Laczko E., Hu J., Wieser K., Meyer D.C. (2017). Genomic and lipidomic actions of nandrolone on detached rotator cuff muscle in sheep. J. Steroid Biochem. Mol. Biol..

[29] Parssinen M., Karila T., Kovanen V., Seppälä T. (2000). The effect of supraphysiological doses of anabolic androgenic steroids on collagen metabolism. Int. J. Sports Med..

[30] Evans N.A. (2004). Current Concepts in Anabolic-Androgenic Steroids. Am. J. Sports Med..

[31] Abate M., Salini V., Andia I. (2016). How Obesity Affects Tendons?.

[32] Bi Y., Ehirchiou D., Kilts T.M., Inkson C.A., Embree M.C., Sonoyama W., Li L., Leet A.I., Seo B.M., Zhang L. (2007). Identification of tendon stem/progenitor cells and the role of the extracellular matrix in their niche. Nat. Med..

[33] Zhang J., Yuan T., Wang J.H. (2016). Moderate treadmill running exercise prior to tendon injury enhances wound healing in aging rats. Oncotarget.

[34] De Mos M., Koevoet W.J., Jahr H., Verstegen M.M., Heijboer M.P., Kops N., Van Leeuwen J.P., Weinans H., Verhaar J.A., van Osch G.J. (2007). Intrinsic differentiation potential of adolescent human tendon tissue: An in-vitro cell differentiation study. BMC Musculoskelet. Disord..

[35] Zhang J., Wang J.-C. (2013). The Effects of Mechanical Loading on Tendons-An In Vivo and In Vitro Model Study. PLoS ONE.

[36] Scott A., Zwerver J., Grewal N., De Sa A., Alktebi T., Granville D.J., Hart D.A. (2015). Lipids, adiposity and tendinopathy: Is there a mechanistic link? Critical review. Br. J. Sports Med..

[37] Ackerman J.E., Geary M.B., Orner C.A., Bawany F., Loiselle A.E. (2017). Obesity/Type II diabetes alters macrophage polarization resulting in a fibrotic tendon healing response. PLoS ONE.

[38] Gaida J.E., Ashe M.C., Bass S.L., Cook J.L. (2009). Is adiposity an under-recognized risk factor for tendinopathy? A systematic review. Arthritis Care Res..

[39] Józsa L., Kannus P. (2007). Histopathological findings in spontaneous tendon ruptures. Scand. J. Med. Sci. Sports.

[40] Egerbacher M., Arnoczky S.P., Caballero O., Lavagnino M., Gardner K.L. (2008). Loss of Homeostatic Tension Induces Apoptosis in Tendon Cells: An In Vitro Study. Clin. Orthop. Relat. Res..

[41] Benson R.T., McDonnell S.M., Knowles H.J., Rees J.L., Carr A.J., Hulley P.A. (2010). Tendinopathy and tears of the rotator cuff are associated with hypoxia and apoptosis. J. Bone Jt. Surg..

[42] Lundgreen K., Lian Ø.B., Engebretsen L., Scott A. (2011). Tenocyte apoptosis in the torn rotator cuff: A primary or secondary pathological event?. Br. J. Sports Med..

[43] Paschoal M., de Cássia Marqueti R., Perez S., Selistre-de-Araujo H.S. (2009). Nandrolone inhibits VEGF mRNA in rat muscle. Int. J. Sports Med..

[44] Petersen W., Unterhauser F., Pufe T., Zantop T., Südkamp N., Weiler A. (2003). The angiogenic peptide vascular endothelial growth factor (VEGF) is expressed during the remodeling of free tendon grafts in sheep. Arch. Orthop. Trauma Surg..

[45] Mujika I., Padilla S. (2000). Detraining: Loss of Training-Induced Physiological and Performance Adaptations. Part I. Sport Med..

[46] Van Roie E., Walker S., Van Driessche S., Baggen R., Coudyzer W., Bautmans I., Delecluse C. (2017). Training load does not affect detraining’s effect on muscle volume, muscle strength and functional capacity among older adults. Exp. Gerontol..

[47] Gondin J., Guette M., Ballay Y., Martin A. (2006). Neural and muscular changes to detraining after electrostimulation training. Eur. J. Appl. Physiol..

[48] Kubo K., Ikebukuro T., Maki A., Yata H., Tsunoda N. (2012). Time course of changes in the human Achilles tendon properties and metabolism during training and detraining in vivo. Eur. J. Appl. Physiol..

[49] Salamanna F., Frizziero A., Pagani S., Giavaresi G., Curzi D., Falcieri E., Marini M., Abruzzo P.M., Martini L., Fini M. (2015). Metabolic and cytoprotective effects of in vivo peri-patellar hyaluronic acid injections in cultured tenocytes. Connect. Tissue Res..

